# The costs of reducing loss to follow-up in South African cervical cancer screening

**DOI:** 10.1186/1478-7547-3-11

**Published:** 2005-11-15

**Authors:** Jeremy D Goldhaber-Fiebert, Lynette E Denny, Michelle De Souza, Thomas C Wright, Louise Kuhn, Sue J Goldie

**Affiliations:** 1 Harvard Initiative for Global Health, Harvard University, Massachusetts, USA; 2 Department of Obstetrics and Gynecology, University of Cape Town, South Africa; 3 Department of Pathology, College of Physicians and Surgeons of Columbia University, New York, USA; 4 Gertrude H. Sergievsky Center, College of Physicians and Surgeons, and Division of Epidemiology, Joseph L. Mailman School of Public Health, Columbia University, New York, USA

## Abstract

**Background:**

This study was designed to quantify the resources used in reestablishing contact with women who missed their scheduled cervical cancer screening visits and to assess the success of this effort in reducing loss to follow-up in a developing country setting.

**Methods:**

Women were enrolled in this Cape Town, South Africa-based screening study between 2000 and 2003, and all had scheduled follow-up visits in 2003. Community health worker (CHW) time, vehicle use, maintenance, and depreciation were estimated from weekly logs and cost accounting systems. The percentage of women who attended their scheduled visit, those who attended after CHW contact(s), and those who never returned despite attempted contact(s) were determined. The number of CHW visits per woman was also estimated.

**Results:**

3,711 visits were scheduled in 2003. Of these, 2,321 (62.5%) occurred without CHW contact, 918 (24.8%) occurred after contact(s), and 472 (12.7%) did not occur despite contact(s). Loss to follow-up was reduced from 21% to 6%, 39% to 10%, and 50% to 24% for 6, 12, and 24-month visits. CHWs attempted 3,200 contacts in 530 trips. On average, 3 CHWs attempted to contact 6 participants over each 111 minute trip. The per-person cost (2003 Rand) for these activities was 12.75, 24.92, and 40.50 for 6, 12, and 24-month visits.

**Conclusion:**

CHW contact with women who missed scheduled visits increased their return rate. Cost-effectiveness analyses aimed at policy decisions about cervical cancer screening in developing countries should incorporate these findings.

## Background

The vast majority of cervical cancer deaths occur among women in developing countries where screening has been largely unavailable [1]. The effectiveness of cervical cancer screening has been demonstrated by the dramatic decline of cervical cancer in developed countries in which programs relying on repeated cervical Pap smears have been successfully implemented [2,3].

Traditionally, conventional cervical cancer screening using cervical cytology requires up to three visits (screening, colposcopy/biopsy, and treatment). In developed country settings, HPV DNA testing has been proposed both as a primary screening test in older women and in conjunction with cervical cytology as triage for equivocal cytologic results [4,5]. Recently, others have proposed using either cervical cytology or HPV DNA testing in two-visit strategies that eliminate confirmation with colposcopy/biopsy prior to treatment, or using one-visit, "see and treat" strategies with visual inspection with acetic acid (VIA) [6-8]. Regardless of the screening test chosen, an important motivation for these alternative strategies is to reduce the screening program's susceptibility to loss to follow-up by reducing the number of visits at which loss to follow-up can occur [9-11].

A number of cost-effectiveness analyses (CEAs) have been conducted, comparing many of these screening strategies in developing country contexts [12-14]. One key finding is that for screening strategies that require women to return to the clinic multiple times, loss to follow-up drives down the effectiveness of screening, regardless of screening modality. Furthermore, the cost of screening accrues as each woman is screened. The primary benefit from screening, the prevention of cervical cancer, is only realized for those women with positive test results and true precancerous lesions who are ultimately treated.

In low resource settings, levels of loss to follow-up in cervical cancer screening programs where follow-up visits were scheduled four weeks or more after the initial visit range from 10% to 70% [10,15-21]. Efforts to reduce loss to follow-up and to maintain it at an acceptably low level are thus a key part of cervical cancer screening programs. Such efforts can be time-intensive and costly and do not guarantee that all women return to the clinic for follow-up. Quantifying the cost and effectiveness associated with achieving acceptably low levels of loss to follow-up is essential for providing an estimate of the investment necessary to achieve cervical cancer screening coverage at the population level as well as for estimating the true cost-effectiveness of cervical cancer screening in these settings.

We used year 2003 data from the Khayelitsha Cervical Cancer Screening Program (KCCSP), a multi-year, South African program, to examine the success of CHW home visits in reducing loss to follow-up as well as the extent and type of resources used in this effort.

## Methods

### Study setting

The Khayelitsha Cervical Cancer Screening Program encompasses a multi-year, multi-site study designed to evaluate the effectiveness of a variety of cervical cancer screening and treatment strategies among a largely poor, black, peri-urban, South African population in a real-world setting. Approximately 25% of participants live in informal settlements without basic services such as electricity and water, and another 45% live in informal settlements with some basic services. The remaining 30% live in formal settlements.

At each scheduled appointment, women are tested with cervical cytology, HPV DNA testing using Hybrid Capture II, and visual inspection with acetic acid. The program has generated data that support the effectiveness and cost-effectiveness of various screening strategies [11,22,23]. In an effort to minimize loss to follow-up, CHWs drive throughout the community to visit women in their homes if they have missed appointments. Home visits by CHWs are used because most participants do not have telephones.

### Study population

All study participants who had appointments in 2003 were considered eligible for inclusion in the main analysis. Study participants had one or more of four different types of appointments scheduled for during this period: 6-month, 12-month, 24-month, and 36-month visits. We restricted our analysis of CHW home visit effectiveness and examined the first three types of appointments because the number of 36-month appointments in 2003 was small (n = 277) relative to 6-month (n = 919), 12-month (n = 1820), and 24-month (n = 980) appointments.

KCCSP was approved by the Institutional Review Boards of Columbia University and the University of Cape Town, and all study participants gave written informed consent.

### Effectiveness

To assess the effectiveness of CHW home visits, we estimated the number of appointments that would have been missed due to loss to follow-up and the percentage of these appointments rescheduled and attended due to home visits. We accounted for the possibility that each participant might have more than one appointment within the period of this study and that CHWs might make more than one home visit per participant.

Participant study number, entries for all scheduled appointments during 2003, and the date(s) on which the participant arrived for these appointments were extracted from the main study database. This data was linked to data transcribed from weekly forms used by CHWs to record participant study numbers and the date of the CHW visits. Using the date of the scheduled appointment, the date of the CHW visit(s), and the date on which the participant actually visited the clinic, the appointment type for which the participant was visited was identified. Using this method, those participants who had been visited by CHW but never returned for their appointments were also identified. Similar estimates subcategorized by appointment type were also generated.

### Costs

To estimate costs, we first identified the different types of resources used in the CHW home visits. Resources included the time the CHWs spent driving to and from as well as visiting participants who had missed appointments, fuel used during the trips to visit participants, and the maintenance and depreciation in the value of the vehicles. Next, the average monetary value for each resource type was estimated. Finally, the estimated quantity of each type of resource was multiplied by its estimated monetary value, and the results were then summed to calculate the total cost of the effort to reduce loss to follow-up.

The quantity of CHW time used for visits was derived from weekly reporting sheets that identified the amount of time spent making home visits each day, the study number of each participant visited, and the CHWs who went on each visit. The value of CHW time was estimated in two ways. First, the salary scale employed within the study was used to estimate the actual cost to the study. Second, South African health worker wage scales were used to estimate the cost of CHW time if such an effort were conducted within the national health system.

The costs of fuel and maintenance for vehicles used by the CHWs to make their visits were extracted from the study's cost accounting system. The system produced monthly reports detailing fuel and maintenance costs for each study vehicle. Since the vehicles were also used for other tasks such as transportation of specimens to laboratories for analysis, only a percentage of the total vehicle costs were truly attributable to the CHW visits. This percentage was calculated as the relative proportion of time that each vehicle was used by the CHWs to make home visits.

Vehicle depreciation was estimated based on the initial purchase price of the vehicles, the expected useful life of each vehicle, and the assumption that the final resale value of the vehicle would be negligible. Straight line depreciation was employed to calculate the total depreciation for one year, using a 3% discount rate [24,25]. The same percentage of fuel and maintenance costs attributable to CHW visits was applied to the total vehicle depreciation cost to calculate vehicle depreciation attributable to CHW visits.

All costs are presented in 2003 South African Rand and do not include any form of tax since taxes represent transfer payments and are not real economic costs [24]. The exchange rate between 2003 South African Rand and 2003 US Dollars was 7.56 Rand per Dollar [26].

### CHW visits

Since it was possible that multiple participants were visited on each CHW trip; that more than one CHW went on each trip; and that some participants received multiple CHW visits, we accounted for this in our estimates using data from the weekly CHW visit logs. To minimize potential bias of the number of CHW visits per participant due to censoring (e.g., additional CHW visits in early 2004 not counted for participants first visited in December of 2003), data on average number of CHW visits per participant was based solely on participants with scheduled appointments in the first half of 2003.

### Cost per appointment

Based upon the total number of CHW home visits conducted and the total cost to carrying out these home visits, a cost per CHW home visit was calculated. Cost per woman screened was calculated by appointment type because the time since the previous visit differed by appointment type: 1) 5 months between the 1-month visit and the 6-month visit; 2) 6 months between the 6-month visit and the 12-month visit; 3) 12 months between the 12-month visit and the 24-month visit. To do this, the ratio of the number of CHW visits conducted for a particular appointment type to the total number of women attending that appointment type was calculated. Then, this ratio was multiplied by the cost per CHW visit to derive the marginal cost per woman for CHW home visits.

### Ranges and sensitivity analyses

Because there was uncertainty in a number of parameters used to estimate total cost, we estimated an upper and lower bound for costs, reflecting a combination of differing assumptions regarding salary and percentage of vehicle costs attributable to CHW home visits. Because it was necessary to infer the appointment type for which CHW home visits were attempting to reduce loss to follow-up, we applied alternate assumptions to generate ranges of estimates of cost per woman screened.

## Results

### Effectiveness of CHW visits

**Figure 1:**
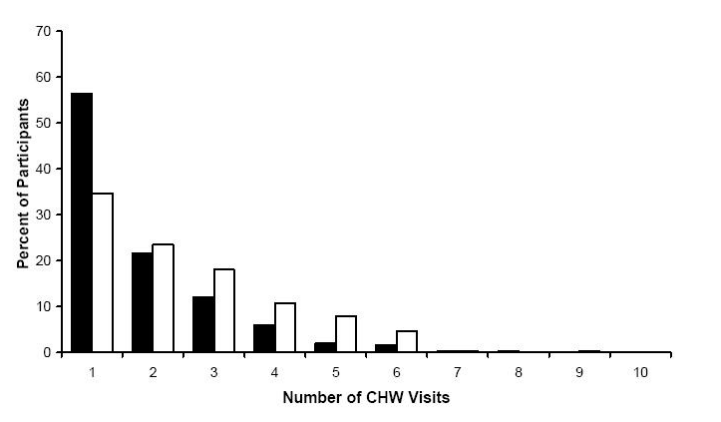
**CHW Visits for Participants with Appointments in the First Half of 2003**. The distribution of the number of CHW home visits for those women who eventually returned for their appointments (black bars) and those women who never returned for their appointments (white bars).

Potential loss to follow-up differs by appointment type as does the success of CHW visits in preventing loss to follow-up. Figure 1 shows the distribution of the number of visits for participants with appointments in the first half of 2003 who returned for their appointments after CHW visits and for those who did not return. For those women who missed their scheduled appointment, the mean number of CHW visits was 1.98 for those with 6-month appointments, 2.06 for those with 12-month appointments, and 2.05 for those with 24-month appointments.

**Figure 2:**
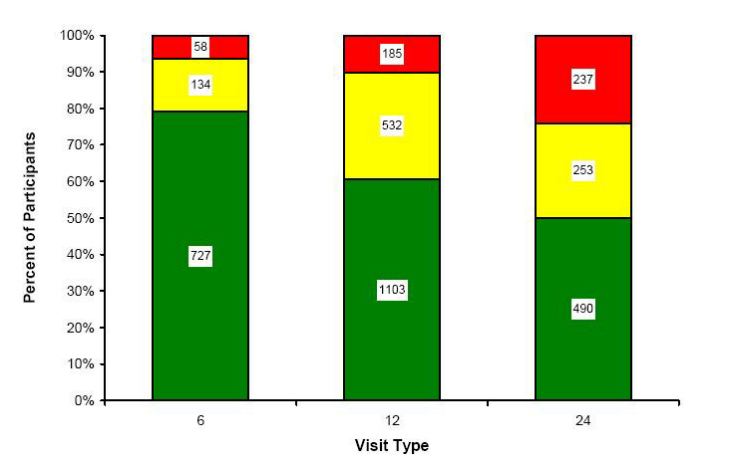
**Appointments Attended by Type and CHW Visit Status**. The number and percentage of women who attended appointments without CHW home visits (green bars), women who attended appointments after CHW home visits (yellow bars), and women who never returned for their appointments (red bars) for each appointment type.

Figure 2 shows the percentage of women who returned without CHW visits, the percentage of women who returned after CHW visits, and those who did not return despite CHW visits. Without CHW visits, loss to follow-up for participants scheduled for 6 month, 12 month, and 24-month visits would have been 21%, 39%, and 50% respectively. With CHW visits, loss to follow-up was reduced to 6%, 10%, and 24% respectively.

### Quantities of resources used

CHW home visits involve groups of CHWs driving into Khayelitsha and surrounding township areas to visit study participants who have not returned to the clinic for their scheduled appointments. For security reasons, CHWs travel in groups of up to four, with larger groups generally going when informal settlements are visited.

In total, CHWs conducted approximately 3,200 visits in 530 trips during 2003. Means for staff per trip, participants visited per trip, time per trip, and time per participant visit were calculated along with their standard deviations from weekly CHW home visit logs (Table 1).

**Table 1:** Community Health Worker Visits to Participants

	**Estimate**	SD
CHWs per trip (trips = 457)*	2.82	0.85
Participants visited per trip (trips = 530)*	5.65	2.46
Time per trip (min) (trips = 386)*	111.32	52.60
Time per participant visited (min) (trips = 386)*	22.09	12.87

* The numbers of trips for these parameters differ because not all information was provided on all weekly report forms. To calculate ratios, only trips for which all information was available were included.

Primary observations of CHWs were conducted on several days to corroborate the information contained in the weekly logs. This observation found average visit time was 20.75 minutes (SD = 8.56, participants = 16). This matched the information from the reports (22.09 minutes, SD = 12.87, trips = 386). Additionally, it was found that approximately 70% of the participant visit time was spent driving between each participant's home.

On the 230 days per year that the project operates, its vehicles are used for three main purposes, staff transport, specimen transport, and CHW visits to participants. Approximately three hours per day are spent on staff and specimen transport. Approximately four hours and 20 minutes are spent on CHW visits per day with 70% of this time spent driving. Thus, 50% of the vehicles' costs are allocated to CHW visits.

### Costs of resources used

Average monthly salaries for CHWs working for the KCCSP are approximately 4000 Rand, while average salaries for CHWs working in South Africa are approximately 2000 Rand per month.

The project operates four vehicles. The purchase price of each vehicle was approximately 40,000 Rand, excluding taxes. The mean and standard deviation of yearly fuel costs per vehicle are 9,111 Rand (SD = 1,735). The mean and standard deviation of yearly maintenance costs are 6,761 Rand (SD = 2,189). Using a 3% discount rate and five year, straight line depreciation, the yearly depreciation cost of each vehicle was 8,684 Rand. Table 2 presents the component costs attributable to CHW visits.

**Table 2:** Component Costs Attributable to CHW Visits (2003 Rand)

	**Estimate**
CHW Time Cost	43,335
Fuel Cost	18,222
Maintenance Cost	13,522
Depreciation Cost	17,368

Total Cost	92,447

### Cost per screened participant

The cost per CHW visit was 28.32 Rand. If the higher study CHW wage of 4,000 Rand per month was used and 80% of CHW visit time was spent driving, the estimate increases to 39.96 Rand per CHW visit. If an even lower, public sector wage of 1,500 Rand per month and 50% of CHW visit time was spent driving, the estimate decreases to 21.14 Rand per CHW visit.

To attribute these costs on a per-screened participant basis, it is necessary to examine the relative number of appointments and relative effectiveness of CHW visits at reducing loss to follow-up for 6, 12, and 24-month appointments. Table 3 shows the results of this analysis. Because of high success in CHW visits leading to participant return, 6-month per participant costs are 12.75 Rand. There is an increasing trend in per participant costs for the 12 and 24-month visits, with per participant cost equaling 24.92 Rand and 40.50 Rand respectively.

**Table 3:** CHW Visit Cost Attribution by Appointment Type (2003 Rand)

	**6-Month Visit**	**12-Month Visit**	**24-Month Visit**
Participants Attending Appointments	861	1,635	743
CHW Visits Conducted	384	1,438	1,062
Visits per Participant	0.45	0.88	1.43
CHW Visit Cost per Participant (base case)	12.75	24.92	40.50
CHW Visit Cost per Participant (lower estimate)	9.51	18.60	30.23
CHW Visit Cost per Participant (upper estimate)	17.98	35.16	57.14

There was some ambiguity in the participant data as to which appointment type a particular CHW visit should be attributed. For example, for a participant with 6 and 12-month appointments within 2003 who missed both appointments and who received 4 CHW visits, it was not always clear which CHW visits led to attendance at each appointment. In the base case, we attributed the total number of CHW visits to both appointments since separate cost per woman estimates were calculated. However, this provides an overestimate of costs.

Two other alternatives were explored. In the first alternative, the earlier appointment was assumed to be the appointment for which all CHW home visits were necessary. In the second alternative, when such an ambiguity existed, the count of CHW visits was divided evenly between the two appointments.

**Figure 3:**
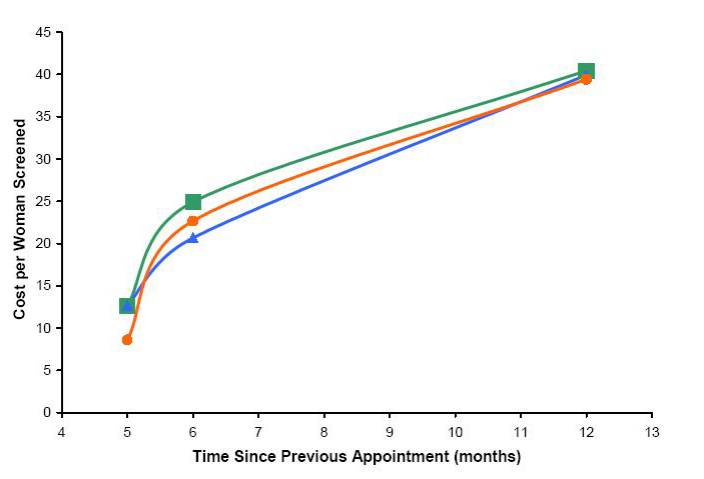
**Cost per Woman Screened by Time since Previous Appointment, Sensitivity Analysis**. The sensitivity analysis shown is of the cost per woman screened for CHW visits using alternative assumptions. The base case (green line and squares) attributes CHW visits to all missed appointments occurring during 2003. Alternative assumption 1 (blue line and triangles) attributes CHW visits to the first missed appointment occurring during 2003. Alternative assumption 2 (orange line and circles) evenly divides CHW visits between all missed appointments occurring during 2003.

Figure 3 shows the CHW home visit cost per woman using different inference rules to assign CHW home visits to the various appointment types. Per-woman screened costs ranged from 8.59–12.62 Rand, 20.65–24.89 Rand, and 39.42–40.45 Rand for 6, 12, and 24-month appointments respectively.

## Discussion

The objective of this study was to estimate the costs and effectiveness associated with an intervention to reduce the loss to follow-up after an initial cervical cancer screening visit in a South African screening program delivered to women of lower socio-economic status. We found that loss to follow-up was reduced significantly by community health worker home visits, and this effect was most pronounced when the intervention occurred in close proximity to the initial appointment. The costs of the CHW intervention were substantial when considered in the context of the total per-woman cost of cervical cancer screening. The CHW home visit costs were 8–15%, 15–29%, and 25–47% of the total per-woman screened cost for 6, 12, and 24-month visits [13].

Ultimately, the costs of cervical cancer screening including efforts to reduce loss to follow-up must be considered in relationship to the level of cervical cancer reduction. In an ethical study conducted in a reasonable time-frame, it would not be possible to measure actual reduction in cervical cancer incidence due to reduction in loss to follow-up. Thus, model-based studies are best suited to use our estimates to refine results from previous cost-effectiveness analyses of cervical cancer screening programs in developing countries. In addition, analysts conducting cost studies for short-term budgetary planning might wish to include these costs to reflect the full range of resources likely to be required in a new cervical cancer screening program.

This study has several limitations. First, we relied on data from a single year derived from sites in one peri-urban township outside of Cape Town. While the areas where participants live are spread over kilometers of land and in the winter rainy seasons, the unpaved roads of the informal settlements become difficult to navigate, the generalizability of these results to other parts of South Africa or, for that matter, to other developing countries is uncertain. Second, the data were derived from CHW home visits connected to a clinical research study. Possible selection biases may operate to make participants more or less likely to miss clinical appointments or to respond to efforts to reduce loss to follow up. Because the staff is better paid and likely better trained than average public sector CHWs, differences in competence and motivation level may also impact the success if this effort were to be replicated elsewhere. Third, other methods of reducing loss to follow-up such as telephone calls, letters, and specifically-targeted preventive educational sessions were not evaluated as alternatives to CHW visits. Because most participants reported not having telephones and because of difficulties in timely and accurate mail deliveries to all participants, we believe that these interventions were less appropriate for reducing loss to follow-up in this setting. An important question remains as to whether other potentially less costly and/or more effective methods exist to reduce loss to follow-up. Finally, the sustainability of this effort over many years and locations has not been tested.

To reflect the variability and uncertainty inherent in delivering cervical cancer screening services we have produced plausible ranges of costs and effectiveness for efforts to reduce loss to follow-up. Such estimates of the costs and effectiveness of reducing loss to follow-up should be considered as programs are planned and scaled-up to national population coverage levels.

## Conclusion

In a South African cervical cancer screening study, loss to follow-up was reduced by CHW visits to women who had not attended their regularly scheduled appointments. The effectiveness of the CHW intervention was higher for appointments scheduled closer to initial screening visits. The total costs associated with CHW visits were appreciable, and the per-woman cost increased as the time between initial appointment and scheduled appointment increased. The cost-effectiveness of preventing loss to follow-up is an important consideration in planning national screening programs in resource poor settings

## List of Abbreviations

CEA: cost-effectiveness analysis

CHW: community health worker

HPV: human papillomavirus

KCCSP: Khayelitsha Cervical Cancer Screening Program

VIA: visual inspection with acetic acid

## Competing interests

The author(s) declare that they have no competing interests.

## Authors' contributions

JGF, LED, and SJG participated in the conception and design of the study. LED and TCW provided study materials or patients. JGF performed the collection and assembly of data as well as the analysis and interpretation. JGF drafted the article, and JGF, LED, MD, LK, and SJG provided critical revision of the article for important intellectual content.
